# Application of Time‐Driven Activity‐Based Costing for Protocol‐Driven Adult Open Airway Reconstruction

**DOI:** 10.1002/ohn.70287

**Published:** 2026-05-10

**Authors:** Andrew D. P. Prince, Fatameh Ramazani, Robbi A. Kupfer, Norman D. Hogikyan, Robert J. Morrison, Pratyusha Yalamanchi

**Affiliations:** ^1^ Department of Otolaryngology–Head and Neck Surgery Michigan Medicine Ann Arbor Michigan USA

**Keywords:** airway reconstruction, healthcare economics, perioperative efficiency, protocolized care, time‐driven activity‐based costing, value‐based care

## Abstract

**Objective:**

To describe a protocol‐driven approach to adult open airway reconstruction for benign airway stenosis and apply time‐driven activity‐based costing (TDABC) methodology to characterize resource utilization and cost drivers.

**Study Design:**

Retrospective cohort study.

**Setting:**

Tertiary academic medical center.

**Methods:**

Adult patients undergoing tracheal or cricotracheal resection (TR/CTR) between 2022 and 2024 were reviewed. Multidisciplinary process mapping was performed using a modified Delphi technique. Validated TDABC methodology was applied across the operative admission. Univariate and generalized linear mixed models were used to examine associations between patient and hospital characteristics and the cost of care delivery.

**Results:**

Twenty patients (70% female, mean age 41 [±14.8] years, and mean Charlson Comorbidity Index 1 ± 1.1) underwent airway reconstruction. TR was most common (n = 13), followed by CTR (n = 5) and revision CTR (n = 2). Protocol‐driven management included clinic evaluation, direct laryngoscopy/bronchoscopy, operative reconstruction, and postoperative intensive care unit (ICU)/floor management. Complications included seroma, tracheitis, and hematoma in one patient. Ten patients required a single post‐reconstruction procedure, six required multiple, and four required none. One patient remained tracheostomy dependent at follow‐up. Total cost of care delivery across admission was $135,598.08 ± 25,100, with ICU care accounting for 60.5% of costs. ICU length of stay and nursing labor were significant cost drivers (*P* < .05), while operating room, supplies, and physician effort accounted for only 10% of total costs.

**Conclusion:**

Protocol‐driven open airway reconstruction is resource‐intensive, with postoperative ICU care representing the primary cost driver. TDABC provides a granular framework for understanding cost structure and identifying opportunities for future value‐based optimization in complex airway surgery.

Laryngotracheal stenosis (LTS) is a debilitating narrowing of the proximal airway, often requiring repeated surgical interventions to manage symptoms such as dyspnea and stridor and to prevent airway obstruction or death.^1^, ^2^ Annual healthcare costs for LTS are estimated at $4080.09 per patient, comparable to chronic conditions such as diabetes mellitus and chronic obstructive pulmonary disease.^3^, ^4^ Yet, the economic burden of both the condition and the related surgical management remains poorly understood.^3^

Surgical options range from various endoscopic procedures to open reconstruction, including tracheal resection (TR) and cricotracheal resection (CTR).^1^, ^2^, ^3^ Data from the North American Airway Collaborative (NoAAC) identify TR/CTR as the most durable interventions for idiopathic subglottic stenosis (SGS), reducing the need for subsequent surgical intervention.^1^ Similar outcomes have been reported for high‐grade iatrogenic LTS.^5^ The mean cost of TR/CTR is reported at $8,583.91 per case by a small cohort study (seven patients) and has relied on hospital charge or reimbursement data, which do not accurately reflect the true costs of care delivery.^3^

Time‐driven activity‐based costing (TDABC), a validated method focused on value‐based care, estimates cost based on the time and resources required for each step in a care pathway.^6^, ^7^ Unlike charge‐based accounting, TDABC not only provides precise cost quantification but also highlights inefficiencies, allowing for streamlined high‐quality care.^6^, ^7^, ^8^

The objective of this study was to develop a protocol‐driven process map for adult open airway reconstruction and apply TDABC to characterize the actual cost and resource utilization associated with TR and CTR for benign airway stenosis.

## Methods

This retrospective cohort study included 20 adult patients who underwent complex airway reconstruction with primary anastomosis (TR/CTR) at a single tertiary academic medical center between August 2022 and August 2024. The study was approved by the University of Michigan Institutional Review Board (HUM00188222).

### Process Mapping and Episode Definition

**Figure 1:**
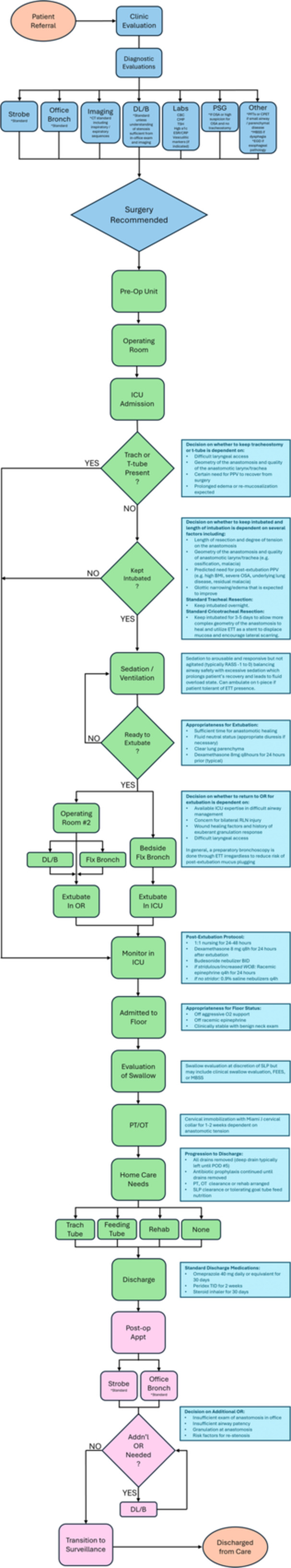
Process map and care protocol. BID, bis in die (twice a day); BMI, body mass index; CBC, complete blood count; CMP, comprehensive metabolic panel; CPET, cardiopulmonary exercise test; CT, computed tomography; DL/B, direct laryngoscopy and bronchoscopy; EGD, esophagogastroduodenoscopy; ESR/CRP, erythrocyte sedimentation rate/C‐reactive protein; ETT, endotracheal tube; FEES, flexible endoscopic evaluation of swallow; ICU, intensive care unit; MBSS, modified barium swallow study; OR, operating room; OSA, obsrtuted sleep apnea; PFT, pulmonary function test; POD, post operative day; PPV, positive pressure ventilation; PSG, polysomnography; PT/OT, physical therapy/occupational therapy; RASS, Richmond Agitation‐Sedation Scale; RLN, recurrent laryngeal nerve; SLP, sleep language and pathology; TID, ter in die (three times daily); TSH, thyroid stimulating hormone; WOB, work of breathing.

A multidisciplinary process map defining the global care period, from preoperative evaluation through postoperative admission, was developed using a modified Delphi technique (Figure 1).^9^, ^10^ We employed validated TDABC methodology across the operative admission for CTR/TR. Preoperative outpatient evaluation and post‐discharge outpatient follow‐up costs were not included due to unavailable data.

Patient demographics, comorbidities, indications for surgery, surgical site and procedure, operative variables, and postoperative outcomes such as inpatient length of stay, complications, and need for reoperation were extracted from the medical chart.

### Time‐Driven Activity‐Based Costing Framework

We followed previously reported methods to apply TDABC to our cohort analysis.^6^ Cost data were obtained through an institutional pilot program launched in 2016 by the Clinical Design and Innovation Program to support care redesign in selected service lines with established outcome tracking and multidisciplinary engagement.^11^ Although derived using institutional TDABC infrastructure, this approach can be replicated using standard hospital staffing models, salary support data, and unit‐level operating costs. TDABC analysis followed a standardized two‐step approach: (1) detailed process mapping to identify all care activities and resources involved in the operative admission; (2) calculation of capacity cost rates (CCRs) for each identified resource.

Operative time, postoperative unit time, and personnel involvement were extracted from electronic medical record case logs and encounter‐specific documentation. Personnel costs were derived from full‐time equivalent salary plus benefits using institutional payroll data. Physician labor costs were estimated using work relative value units (wRVUs) for relevant Current Procedural Terminology (CPT) codes, as direct time‐based physician documentation was not consistently available. Supply costs were obtained from institution‐specific chargemaster pricing, as true acquisition costs were not available; this approach was applied uniformly across cases to permit relative cost comparisons within the TDABC framework.

### Capacity Cost Rate Definition

CCRs were calculated by dividing the total annual cost of each resource by its practical capacity, expressed in minutes of availability, consistent with standard TDABC methodology.^6^, ^9^, ^10^, ^12^, ^13^, ^14^ Resource costs incorporated both fixed and variable components. Indirect costs such as maintenance, utilities, and administrative support were included within care‐setting–specific CCRs (eg, operating room [OR], intensive care unit [ICU], and inpatient floor). Hospital‐wide operating costs not attributable to a specific care unit were allocated as overhead using a separate CCR.

As an example, the ICU cost per minute represents the ICU CCR, defined as the combined unit cost inclusive of all fixed and variable resources identified through ICU process mapping. This CCR incorporates continuous staffing based on standard 1:2 nurse‐to‐patient ratios, respiratory therapy support, and allocated unit‐level facility overhead. However, in the ICU, the CCR reflects cost per staffed‐bed minute based on practical capacity rather than continuous 24‐hour patient occupancy; cost‐bearing time was derived from fully staffed 8‐hour shifts, consistent with TDABC methodology. At our institution, labor accounted for approximately 60% of ICU costs, consistent with prior reports attributing 50% to 75% of ICU costs to staffing.^15^, ^16^

All CCRs were institution‐derived and reflect modeled inpatient costs rather than external reimbursement, charges, or national benchmarks. Costs were adjusted for inflation using the Consumer Price Index published by the US Bureau of Labor Statistics.

Total inpatient cost of care delivery for each patient was calculated as the sum of all resource costs, determined by multiplying the CCR for each resource by the duration of its use during the inpatient encounter, which is reflected in daily labor staffing (8‐ or 12‐hour shifts) that determine bed utilization and occupancy, and overhead expenses.

### Statistical Analysis

Descriptive statistics were summarized using frequencies for categorical variables and means with standard deviations for continuous variables. Cost data were right‐skewed; however, because high‐cost observations reflect meaningful clinical events in complex surgical care, costs were not transformed or excluded. Associations between patient and operative factors and total inpatient cost of care delivery were evaluated using univariate analyses and generalized linear mixed models. Resource‐level cost contributions were illustrated using a Pareto chart. All analyses were performed using SPSS version 31 (IBM Corp.).

## Results

The study population (n = 20) was 70% female with a mean age 41.7 ± 14.8 years at the time of surgery (Table 1). The study cohort was relatively healthy with a mean and median Charlson Comorbidity Index of 1 ± 1.12, and only 15% ever smoked tobacco. Etiologies for airway stenosis were prolonged intubation (n = 7), previous tracheostomy (n = 4), a combination of both (n = 6), and idiopathic stenosis (n = 3). TR was the most common procedure (n = 13), followed by CTR (n = 5) and revision CTR (n = 2) depending on the level of the stenosis and necessary surgical maneuvers.

**Table 1:** Patient Characteristics

Characteristic		Value
Age, mean (SD)		41.65 (14.78)
Body mass index, mean (SD)		32.25 (7.81)
Charlson Comorbidity Index, mean (SD)		1 (1.12)
Gender, n (%)	Male	6 (30%)
	Female	14 (70%)
Race, n (%)	Caucasian	16 (80%)
	African American	2 (10%)
	Middle Eastern	1 (5%)
	Other	1 (5%)
Ethnicity, n (%)	Non‐Hispanic	19 (95%)
	Hispanic	1 (5%)
Smoking status, n (%)	Ever	3 (15%)
	Never	17 (85%)
Indications for surgery, n (%)	Idiopathic	3 (15%)
	Tracheostomy	4 (20%)
	Prolonged intubation	7 (35%)
	Tracheostomy after prolonged intubation	6 (30%)
Surgery type, n (%)	Cricotracheal resection	5 (25%)
	Revision cricotracheal resection	2 (10%)
	Tracheal resection	13 (65%)

In terms of intraoperative variables, median case length, defined as total intraoperative minutes, was 400 minutes (±132.9) (Table 2). Primary anastomosis was performed in every case, suprahyoid muscular release in 60%, and pretracheal plane release in 95%.

**Table 2:** Intraoperative Variables and Postoperative Outcomes

Characteristic		Value
Primary anastomosis, n (%)	Yes	20 (100%)
	No	0 (0%)
Suprahyoid myotomy, n (%)	Yes	12 (60%)
	No	8 (40%)
Pretracheal release, n (%)	Yes	19 (95%)
	No	1 (5%)
Complication, n (%)	Hematoma	2 (10%)
	Chipped tooth	1 (5%)
	Tracheitis	1 (5%)
	Seroma	1 (5%)
Tracheostomy at most recent follow‐up, n (%)	Yes	1 (5%)
	No	19 (95%)
Intraoperative minutes, median (SD)		400 (132.89)
Length of stay, median (SD)		7.7 (1.9)
LACE score, mean (SD)		5.75 (2.20)
Return to OR, n (%)		1 (5%)
Readmission <30 d after discharge, n (%)		1 (5%)
ER visit <30 d after discharge, n (%)		2 (10%)
Patients initiated phone calls <30 d after discharge, median (SD)		2 (2.54)

Abbreviations: ER, emergency room; LACE, length of stay, acuity, comorbidity, emergency visits; OR, operating room.

Postoperative outcomes included a median postoperative length of stay of 7.7 days (±1.9) and a median ICU stay of 4 days (±2.28). Five patients were immediately extubated postoperatively, and 14 patients remained intubated postoperatively, with a median intubation time of 2.5 days (±2.08). Two patients were selected to return to the OR for extubation based on intraoperative factors (Figure 1). One patient maintained their tracheostomy postoperatively due to anastomotic safety concerns from a calcified trachea. That patient is the only one to require tracheostomy at the most recent follow‐up due to continued tracheal stenosis on post operative day 685, despite multiple endoscopic interventions. No patients required emergent reintubation. One patient required a return to the OR twice for a hematoma, on postoperative day 1 and then again on postoperative day 10. Other complications postoperatively included seroma, cracked tooth, and tracheitis in one patient each. Two patients returned to the emergency department for a dislodged nasogastric feeding tube and the above‐listed hematoma. The patient with the hematoma was the only patient readmitted within 30 days after discharge. Within 30 days after discharge, there was a median of 2 (±2.5) phone calls per patient related to postoperative care. Ten patients required a single post‐reconstruction DLB (±endoscopic dilation, steroid injection, laser), six required multiple, and four required none. No patients underwent an open airway revision surgery or postoperative tracheostomy.

**Figure 2:**
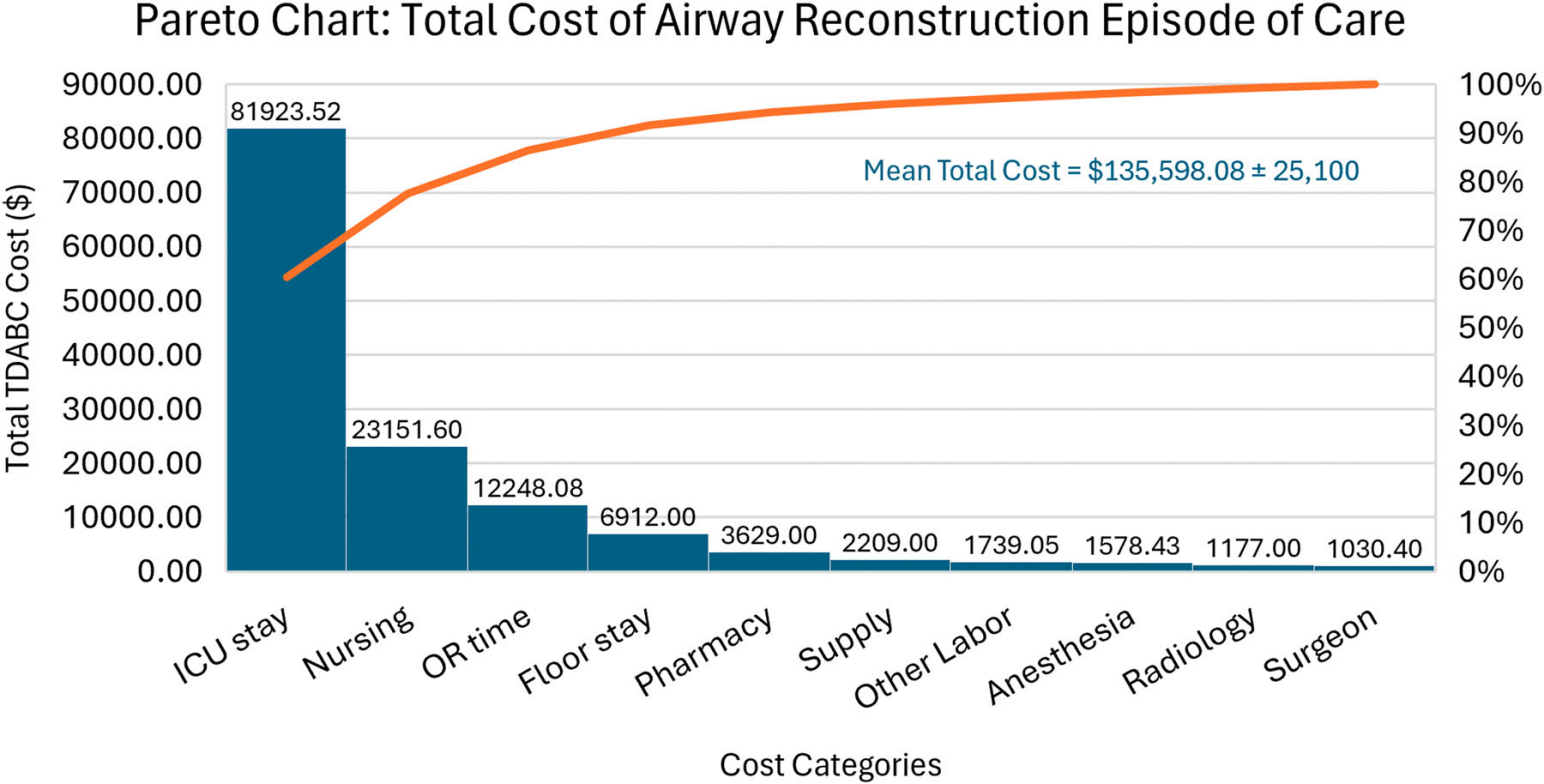
Pareto chart of cost contribution. Demonstrates the relative cost contribution of each resource as a % of the total mean cost of complex airway reconstruction. ICU, intensive care unit; OR, operating room; TDABC, time‐driven activity‐based costing.

CCR for each resource identified in process mapping was calculated (Supplemental Table S1, available online). Notable CCR includes the use of the OR at $30.88 per minute and ICU at $50.81 per minute when adjusted for inflation. Total cost of care delivery across admission was $135,598.08 ± 25,100 with ICU care accounting for 60.5% of costs. A generalized mixed model identified significant cost drivers including length of stay in ICU and nursing labor (*P* < .05). Sensitivity analysis demonstrates that a single day variation from ICU level care to floor involves a mean change of $34,220. The OR time, supplies, and physician effort only accounted for 10% of total costs. Relative cost contributions of each resource are illustrated in the Pareto chart (Figure 2). The total cost breakdowns per category are in Supplemental Table S2, available online. Traditional global billing charges demonstrated a significant difference between the mean cost of CTR $191,070.65 ± 56,033.07 and TR $143,003.93 ± 38,793.88 (*P* = .032). This methodology could not identify costs of individual resources, such as labor, or care setting.

## Discussion

This study presents a standardized approach for management of adult open airway reconstruction, including TR and CTR, and applies TDABC to evaluate resource utilization and cost drivers. In our cohort of 20 patients, the mean cost of care was $135,598.08 ± 25,100. This exceeds previously reported estimates of $8,583.91 per procedure, which were derived from claims‐based reimbursement data rather than institution‐level cost accounting.^3^ Unlike prior studies, which rely on reimbursement data or patient charges, TDABC allows for a granular assessment of actual resource consumption across the entire episode of care.

Using traditional cost accounting for the global billing period, there was a mean difference of $55,000 for CTR and $8,000 for TR compared to TDABC. The increased cost compared to TDABC is likely due to the complete billing period captured. CTR charging is likely higher due to longer intubation and ICU use. This traditional charge‐based costing methodology accounts for reimbursed aspects of care alone and fails to offer a granular characterization of complex care. The TDABC methodology highlights ICU and nursing care as the primary cost drivers, with ICU time costing $50 per minute, nearly twice the cost of the OR ($30 per minute). The median ICU stay was 4 days, and most patients remained intubated for an average of 2.5 days postoperatively. Given ICU shifts of 8‐hour staffing models per day, which in turn determine daily occupancy, sensitivity analysis demonstrated that a single day variation from ICU level care to floor involves a mean change of $34,220. These findings suggest that the timings of extubation and ICU discharge are key targets for cost containment. However, efforts to accelerate ICU throughput must be balanced with patient safety, as premature extubation may lead to adverse outcomes, including airway obstruction, traumatic reintubation, prolonged ICU stay, surgical failure, or death.^17^ We found OR time, physician labor, and OR supplies accounted for less than 10% of total costs, indicating that strategies focused solely on operative efficiency may have a limited impact on overall expenditures. This is different from our prior study of head and neck free tissue transfer, in which operative time and surgical labor were dominant cost drivers; ICU utilization was the primary driver in airway reconstruction, underscoring that cost‐reduction targets are procedure‐specific.^6^ Nonetheless, efforts to reduce operative times, such as improved surgical workflow or optimized team coordination, remain worthwhile and may indirectly reduce downstream resource use.

Developing the clinical process map highlighted challenges intrinsic to rare, high‐complexity operations. Due to low procedural volumes, even among experienced surgeons, there is a limited opportunity for prospective, large‐scale, comparative studies.^18^ Consequently, existing literature is largely retrospective and based on single‐institution experiences.^19^, ^20^ Our protocol was informed by available evidence, local infrastructure, expert opinion, and clinical experience, acknowledging institutional variability and the absence of high‐quality comparative data. While this analysis leveraged institutional TDABC support, similar modeling can be performed using departmental process mapping and hospital finance data, with the primary barrier being analytic time rather than unique infrastructure.

Most components of our protocol are grounded in evidence‐based practices, including the use of peri‐extubation steroids^21^ and anti‐reflux medications.^22^ However, certain aspects remain understudied, most notably, the timing of postoperative extubation, which emerged as a primary cost driver in our analysis. Prior to this study cohort, we favored intraoperative extubation for TR patients. Following early postoperative complications, including air leaks, drain replacements, and anastomotic revisions due to granulation tissue, we transitioned to a more conservative strategy of overnight intubation, aligning with practices commonly used in thoracic surgery. Since implementing this change, air leaks have occurred only in the setting of underlying anastomotic compromise rather than premature extubation. While causality cannot be definitively established, this shift has coincided with a reduction in extubation‐related complications. Ongoing protocol refinement will include consideration of emerging sedation strategies and postoperative care models that have been shown to reduce delayed extubation and ICU length of stay without compromising patient outcomes.^23^

Given mounting pressure on healthcare resource allocation, an improved understanding of cost structure in complex surgical care is increasingly important. TDABC offers a robust framework for quantifying institution‐derived costs in high‐acuity otolaryngology settings by identifying resource‐intensive phases of care and dominant cost drivers. In this study, TDABC was used to enhance cost transparency and inform cost‐conscious clinical and administrative decision‐making rather than to perform a formal assessment of value. Clinical outcomes were not incorporated into a cost outcome equation, and outpatient, indirect, and societal costs were not captured. Accordingly, these findings should be interpreted as foundational inpatient cost data that may support future investigations integrating outcomes and longer‐term costs to more comprehensively evaluate value in complex airway reconstruction.

This study is limited by its single‐center design, which may affect generalizability due to regional differences in labor costs and institutional practices. Supply costs were estimated using chargemaster values rather than acquisition costs, which may overestimate absolute supply expense but is unlikely to alter identification of the primary cost drivers. Additionally, physician costs were estimated via RVUs rather than direct time‐based measurements, and outpatient follow‐up costs were not included. Patient heterogeneity and institutional resources also impact the feasibility of standardizing protocols. Finally, outpatient costs remain unaccounted for in this analysis but are likely to be impactful and warrant future study. Despite these limitations, the methodology and identification of key cost drivers are applicable across institutions and may inform broader efforts toward value‐based care in airway reconstruction.

## Conclusion

We present a comprehensive, protocol‐driven process map for the management of complex airway reconstruction. By applying TDABC methodology, we provide a granular analysis of institution‐derived inpatient costs across the operative admission, revealing substantial discrepancies from traditional charge‐based accounting. The majority of costs were concentrated in postoperative care, particularly ICU utilization. It is critically important for institutions to recognize the resource intensity and be prepared to support high‐acuity postoperative management. These findings highlight the importance of aligning clinical protocols with resource‐intensive phases of care while recognizing that outpatient, indirect, and societal costs were not captured. TDABC offers a practical framework for identifying cost drivers and informing future efforts to optimize value in complex airway reconstruction.

## Author Contributions

**Andrew D. P. Prince**, conceptualization, methodology, investigation, formal analysis, writing—review and editing, visualization; **Fatameh Ramazani**, conceptualization, writing—review and editing,; **Robbi A. Kupfer**, conceptualization, methodology, writing—review and editing; **Norman D. Hogikyan**, conceptualization, methodology, writing—review and editing, supervision; **Robert J. Morrison**, conceptualization, methodology, writing—review and editing, supervision; **Pratyusha Yalamanchi**, conceptualization, methodology, writing—review and editing, supervision.

## Disclosures

### Competing interests

None.

### Funding source

None.

## Supporting information

Supporting File

Supporting File

## References

[1] Gelbard A , Anderson C , Berry LD , et al. Comparative treatment outcomes for patients with idiopathic subglottic stenosis. JAMA Otolaryngol Head Neck Surg. 2020;146(1):20‐29. 31670805 10.1001/jamaoto.2019.3022 PMC6824232

[2] Tierney WS , Huang LC , Chen SC , et al. Comparative treatment outcomes for idiopathic subglottic stenosis: 5‐year update. Otolaryngol Head Neck Surg. 2023;168(6):1570‐1575. 36939627 10.1002/ohn.190

[3] Yin LX , Padula WV , Gadkaree S , et al. Health care costs and cost‐effectiveness in laryngotracheal stenosis. Otolaryngol Head Neck Surg. 2019;160(4):679‐686. 30481123 10.1177/0194599818815068 PMC6443425

[4] Shah RK , Lander L , Choi SS , Zalzal GH . Resource utilization in the management of subglottic stenosis. Otolaryngol Head Neck Surg. 2008;138(2):232‐241. 10.1016/j.otohns.2007.10.015 18241722

[5] Fiz I , Filauro M , Sampieri C , et al. Analysis of complications in (crico‐) tracheal resection anastomosis in adults: a multicenter study. Laryngoscope. 2023;133(11):2910‐2919. 36883671 10.1002/lary.30635

[6] Yalamanchi P , Marentette LJ , Fendrick AM , et al. Application of time‐driven activity‐based costing for head and neck microvascular free flap reconstruction. Otolaryngol Head Neck Surg. 2024;171(1):73‐80. 38643408 10.1002/ohn.739

[7] Keel G , Savage C , Rafiq M , Mazzocato P . Time‐driven activity‐based costing in health care: a systematic review of the literature. Health Policy. 2017;121(7):755‐763. 28535996 10.1016/j.healthpol.2017.04.013

[8] Liu AQ , Allenby S , Lee J , Lea J , Westerberg BD . Time‐driven activity based costing of an annual Canadian cochlear implant program. Otolaryngol Head Neck Surg. 2025;172(2):596‐605. 39308153 10.1002/ohn.977 PMC11773431

[9] McLaughlin N , Burke MA , Setlur NP , et al. Time‐driven activity‐based costing: a driver for provider engagement in costing activities and redesign initiatives. Neurosurg Focus. 2014;37(5):E3. 10.3171/2014.8.FOCUS14381 25363431

[10] Balakrishnan K , Goico B , Arjmand EM . Applying cost accounting to operating room staffing in otolaryngology: time‐driven activity‐based costing and outpatient adenotonsillectomy. Otolaryngol Head Neck Surg. 2015;152(4):684‐690. 25623288 10.1177/0194599814568273

[11] Quality department introduces new internal website. Michigan Medicine Headlines. 2022; https://mmheadlines.org/2018/03/quality-departmentintroduces-new-internal-website/

[12] Kaplan RS , Witkowski M , Abbott M , et al. Using time‐driven activity‐based costing to identify value improvement opportunities in healthcare. J Healthc Manag. 2014;59(6):399‐412. 25647962

[13] French KE , Albright HW , Frenzel JC , et al. Measuring the value of process improvement initiatives in a preoperative assessment center using time‐driven activity‐based costing. Healthc (Amst). 2013;1(3‐4):136‐142. 26249782 10.1016/j.hjdsi.2013.07.007

[14] Crott R , Lawson G , Nollevaux MC , Castiaux A , Krug B . Comprehensive cost analysis of sentinel node biopsy in solid head and neck tumors using a time‐driven activity‐based costing approach. Eur Arch Otrhinolaryngol. 2016;273(9):2621‐2628. 10.1007/s00405-016-4089-z 27170361

[15] Bruyneel A , Larcin L , Martins D , Van Den Bulcke J , Leclercq P , Pirson M . Cost comparisons and factors related to cost per stay in intensive care units in Belgium. BMC Health Serv Res. 2023;23(1):986. 37705056 10.1186/s12913-023-09926-2 PMC10500739

[16] Wunsch H , Gershengorn H , Scales DC . Economics of ICU organization and management. Crit Care Clin. 2012;28(1):25‐37, v. 22123097 10.1016/j.ccc.2011.09.004 PMC3665001

[17] Gupta P , Tobias J , Goyal S , et al. Perioperative care following complex laryngotracheal reconstruction in infants and children. Saudi J Anaesth. 2010;4(3):186‐196. 21189858 10.4103/1658-354X.71577 PMC2980667

[18] Gelbard A , Francis DO , Sandulache VC , Simmons JC , Donovan DT , Ongkasuwan J . Causes and consequences of adult laryngotracheal stenosis. Laryngoscope. 2015;125(5):1137‐1143. 25290987 10.1002/lary.24956 PMC4562418

[19] Dewan K , Berke GS , Chhetri DK . Lessons learned from open laryngotracheal airway resection and primary anastomosis in high risk patients. PLoS One. 2020;15(9):e0238426. 32956400 10.1371/journal.pone.0238426 PMC7505588

[20] Dwyer CD , Qiabi M , Fortin D , et al. Idiopathic subglottic stenosis: an institutional review of outcomes with a multimodality surgical approach. Otolaryngol Head Neck Surg. 2021;164(5):1068‐1076. 33048608 10.1177/0194599820966978

[21] Kuriyama A , Umakoshi N , Sun R . Prophylactic corticosteroids for prevention of postextubation stridor and reintubation in adults. Chest. 2017;151(5):1002‐1010. 28232056 10.1016/j.chest.2017.02.017

[22] Tawfik KO , Houlton JJ , Compton W , Ying J , Khosla SM . Laryngotracheal reconstruction: a ten‐year review of risk factors for decannulation failure. Laryngoscope. 2015;125(3):674‐679. 25491233 10.1002/lary.24963

[23] Thottam PJ , Gilliland T , Ettinger N , Baijal R , Mehta D . Outcomes using a postoperative protocol in pediatric single‐stage laryngotracheal reconstruction. Ann Otol Rhinol Laryngol. 2021;130(8):861‐867. 30767561 10.1177/0003489419830107

