# Supplementary material for: Application of Time‐Driven Activity‐Based Costing for Protocol‐Driven Adult Open Airway Reconstruction
Source: Otolaryngol Head Neck Surg. 2026 May 10;175(2):427–33. doi: 10.1002/ohn.70287 (PMC13418065; doi:10.1002/ohn.70287)
Supplement: Supplementary file 1 — Supporting File [file OHN-175-427-s001.docx]

Supplemental Table 1: Capacity Cost Rates

| Cost Category | Capacity Cost Rate ($/minute) |
| --- | --- |
| Survival Flight | 129.60 |
| Cardiac Procedure Unit | 108.82 |
| Overhead | 56.86 |
| ED | 61.20 |
| Medical Procedure Unit | 46.29 |
| Intensive Care Unit | 14.24 |
| Nursing | 36.94 |
| Operating Room | 30.88 |
| Physician Labor | 22.14 |
| Speech Pathology | 16.87 |
| Pathology | 6.50 |
| Anesthesia | 5.16 |
| Radiology | 4.05 |
| Physical Therapy/Occupational Therapy | 3.94 |
| Ambulatory Care Services | 2.96 |
| Respiratory | 2.90 |
| Nutrition | 2.70 |
| Social Work | 2.65 |
| Virtual Health | 1.21 |
| Pharmacy | 0.86 |
