# Supplementary figures and images for: Application of Time‐Driven Activity‐Based Costing for Protocol‐Driven Adult Open Airway Reconstruction
Source: Otolaryngol Head Neck Surg. 2026 May 10;175(2):427–33. doi: 10.1002/ohn.70287 (PMC13418065; doi:10.1002/ohn.70287)

Supplemental Table 2: Cost Breakdown Per Category


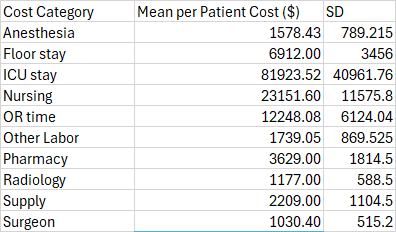

Supplement: Supplementary file 2 — Supporting File [file OHN-175-427-s002.docx]
